# Editorial: Mathematical modeling in discovery and analysis of immune responses

**DOI:** 10.3389/fimmu.2025.1760985

**Published:** 2025-12-16

**Authors:** Penelope A. Morel, Jaroslaw Smieja, Urszula Foryś

**Affiliations:** 1 Department of Immunology, University of Pittsburgh, Pittsburgh, PA, United States; 2 Department of Systems Biology and Engineering, Silesian University of Technology, Gliwice, Poland; 3 Institute of Applied Mathematics and Mechanics, Faculty of Mathematics, Informatics and Mechanics, University of Warsaw, Warsaw, Poland

**Keywords:** autoimmunity, cancer, immunology, infectious disease, mathematical model

Mathematical modeling is an important tool that facilitates formulation and initial testing of hypotheses concerning properties of biological systems, mechanisms controlling their behavior, and novel therapy protocols as well as support analysis of the increasingly large and complex datasets containing experimental and clinical results. The models employed might take various forms – from relatively simple statistical formulae, through black-box models (including AI-based), agent based models, ordinary- and partial differential equations to multilevel models that combine multiple different approaches.

Immunology is a field where such modeling may be particularly helpful, taking into account intertwined mechanisms involved in organism responses to viral or bacterial infections, autoimmune diseases, and immunotherapies. For example, mathematical models can be used by immunologists both to explore theoretical ideas such as kinetic proofreading of TCR activation, and as a tool to analyze the increasingly large and complex datasets that are now being produced. Increasingly, experimental immunologists are working closely with modelers and computer scientists to develop deeper insights into their data, and to design the next set of experiments. New machine learning and other tools can extract not only statistically significant differences between different scenarios but provide additional mechanistic insight which can then be tested experimentally. In this Research Topic, 12 original research articles cover many different applications of mathematical modeling to both the analysis of experimental datasets and the development of new theoretical insights. The topics covered include theoretical analyses of immune cell and antibody function, novel analysis tools for high density datasets and are applied to cancer, autoimmunity and infectious disease.

Three of the articles provide novel theoretical models to understand various aspects of antibody function and TCR binding specificity. The first describes a model that addresses the fact that Antibody Dependent Cellular Cytotoxicity (ADCC) and Antibody Dependent Cellular Phagocytosis (ADCP) are mediated by IgG binding to two different FcγRs expressed on two different cell types (Shoffner-Beck et al.). The ODE model was used to predict immune complexes binding to the ADCC-mediating FcγRIIIA or the ADCP-mediating FcγRIIA as a function of IgG, antigen and FcγR concentration. The model was further used to simulate the effect of a boost of the IgG response in a cohort of individuals in an HIV vaccine trial. Work by Kovacs et al. proposes the use of a thermodynamic titer by applying the Richards function as a more accurate measure of serum antibody titer. The third article in this section examines the impact of feature selection techniques in enhancing predictions of peptide binding to specific TCRs (Teimouri et al.). The analysis shows that using these techniques can improve the ability to predict precise peptide TCR interactions.

The immune response and disease outcome to SARS-CoV-2 infection was the subject of three articles. In one of these studies the authors compared the immune responses to mRNA and adenovirus (AdV) vaccines (Hodgson et al.). Based on data from a cohort of vaccinated health care workers a kinetic model of humoral immunity was constructed that included the dynamics of multiple immune markers. The model predicted higher levels of proliferating memory B cells in individuals receiving the mRNA vaccine and also suggested that increasing the time between the first and second vaccine doses beyond 28 days boosted the production of neutralizing antibody. In the second article the levels of anti-SARS-CoV-2 antibodies were examined in a cohort of patients in Quzhou, China (Yin et al.). The impact of infection, vaccination and hybrid immunity on the antibody titers was examined using multivariate analysis and a decision tree modeling approach. This study identified factors that can inform vaccine strategies and public health measures in the control of COVID-19 disease. Another study tested different machine learning approaches to predict the risk of mortality in older patients with COVID-19 disease, based on blood markers of inflammation and hypercoagulability (Zhu et al.). Nine different machine learning models were used and the study found that the Gaussian naïve Bayes model performed the best in predicting mortality and identified five blood markers that were significantly associated with death in this cohort of 199 patients.

Two studies used mathematical modeling to study the impact of immunotherapy in two different cancer models. CAR-T cell therapy has proved efficacious in liquid hematological malignancies but has limited impact in solid tumors. The first study developed mathematical models to investigate the treatment dynamics of glioblastoma with CAR-T therapy (Szafranska-Leczycka et al.). The model incorporated important biological processes such as tumor growth, CAR-T cell proliferation, time delay of immune reaction, and resistance mechanisms. Simulations were based on existing clinical trial strategies and examined the impact of single or multiple dose approaches. Results indicated that cyclic CAR-T therapy was superior to a single dose and provided insights into the relapse dynamics and the durability of the therapeutic effect. The second article examined the role of tumor infiltrating (TIL) B cells in lung adenocarcinoma and the response to immunotherapy using machine learning tools to analyze pre-existing multiomic datasets (Fang et al.). By analyzing scRNA-seq datasets the study identified a Biomarker-based Risk Index (BRI) based on 27 variables that could be used to predict outcomes in lung cancer patients. Patients with a high BRI were found to have poorer outcomes and reduced response to immune checkpoint blockade. Low BRI was associated with increased tumor infiltration of B cells, CD4+ T cells, NK cells, M2 macrophages and Treg cells. Experimental validation of these results will be needed to substantiate the use of this index.

Three studies examined immune parameters in patients with autoimmune disease using mathematical modeling approaches. One article describes a novel pseudotemporal-based single-cell network inference algorithm, ONIDsc, designed to identify immunopathogenic mechanisms in SLE using ChIP-chip and ChIP-seq datasets from healthy controls and SLE patients (Tejero et al.). This method is based on a previous model, SINGE, and was shown to outperform SINGE and other similar models in identifying immune networks important in SLE. The method identified networks related to innate and B cell activation and identified four genes that were uniquely expressed in SLE patients. Another study examined the development of a two-branch Bayesian network to analyze Raman spectroscopy data in patients with SLE, leading to improvements in diagnostic accuracy (Xu et al.). The use of Raman spectroscopy has been limited by the challenges due to spectral overlap and challenges in identifying relevant features. The authors present a new deep learning approach that can be used to accurately deconvolute Raman spectroscopy data and provide a tool for the diagnosis of SLE and to monitor disease activity. A study examined the responses to TNF-α in PBMCs from healthy donors and RA patients using a single cell multi-omics Cite-seq approach (Perik-Zavodskii et al.). This study revealed that classical monocytes responded the most to TNF-α and this was correlated with increased expression of TNFR2. RA patients showed a more blunted response to TNF-α and this was correlated with reduced TNFR2 expression on classical monocytes.

The final paper in this Research Topic develops a mathematical framework to analyze human neutrophil differentiation states from single cell data (Wigerblad et al.). The study utilized datasets from single cell RNA-seq data from adult human neutrophils. These data were used to inform a novel ODE-based mathematical framework which identifies 5 different transcriptional states in circulating neutrophils from healthy individuals. The model provides a quantitative model of neutrophil transition states that can be used in samples from patients with different diseases.

This Research Topic thus covers a wide range of topics and highlights the myriad ways that mathematical models and analysis tools are impacting the progress of immunological research. As we develop more sophisticated and multi-omic experimental methodologies there will be an increasing need for the development of more tools such as the ones described here.

